# The final communication

**DOI:** 10.1017/S1478951525000367

**Published:** 2025-06-24

**Authors:** Zhaohui Su

**Affiliations:** 1 Key Laboratory of Environmental Medicine Engineering, Ministry of Education, School of Public Health, Southeast University, Nanjing, China; 2 School of Public Health, Southeast University, Nanjing, China

Delirious talks
Darkening faces
Unsettling moans
Stormy cries

Even the air is elegiac
As if the end can be sensed

Fixating glances
Impossible squeezes
Enigmatic gestures
Almost words

If only silence can reveal
The true intentions below

Cascading sighs
Upsetting commotions
Uninitiated passers-by
Confrontational sunrise

Go, things will be fine
Move on fast, we will be fine

## Data Availability

Data are available upon reasonable request.

